# Utilization of Thermally Treated SiC Nanowhiskers and Superplasticizer for Cementitious Composite Production

**DOI:** 10.3390/ma14154062

**Published:** 2021-07-21

**Authors:** Nagilla Azevedo, José Andrade Neto, Paulo de Matos, Andrea Betioli, Maciej Szeląg, Philippe Gleize

**Affiliations:** 1 Laboratory of Application of Nanotechnology in Civil Construction (LabNANOTEC), Department of Civil Engineering, Federal University of Santa Catarina (UFSC), Florianópolis 88040-900, Brazil; nagilla.azevedo@posgrad.ufsc.br (N.A.); paulo.matos@ufsm.br (P.d.M.); p.gleize@ufsc.br (P.G.); 2 Department of Civil Engineering, Federal University of Rio Grande do Sul (UFRGS), Porto Alegre 90035-190, Brazil; jose.andrade@ufrgs.br; 3 Coordenadoria Acadêmica, Federal University of Santa Maria (UFSM), Cachoeira do Sul 96503-205, Brazil; 4 Academic Department of Civil Construction, Federal Institute of Santa Catarina (IFSC), Florianópolis 88020-300, Brazil; andrea.betioli@ifsc.edu.br; 5 Faculty of Civil Engineering and Architecture, Lublin University of Technology, 20-618 Lublin, Poland

**Keywords:** SiC nanowhisker, cementitious composite, rheology, hydration, mechanical properties, microstructure

## Abstract

Nanomaterials are potential candidates to improve the mechanical properties and durability of cementitious composites. SiC nanowhiskers (NWs) present exceptional mechanical properties and have already been successfully incorporated into different matrices. In this study, cementitious composites were produced with a superplasticizer (SP) and 0–1.0 wt % SiC NWs. Two different NWs were used: untreated (NT-NW) and thermally treated at 500 °C (500-NW). The rheological properties, cement hydration, mechanical properties, and microstructure were evaluated. The results showed that NWs incorporation statistically increased the yield stress of cement paste (by up to 10%) while it led to marginal effects in viscosity. NWs enhanced the early cement hydration, increasing the main heat flow peak. NWs incorporation increased the compressive strength, tensile strength, and thermal conductivity of composites by up to 56%, 66%, and 80%, respectively, while it did not statistically affect the water absorption. Scanning electron microscopy showed a good bond between NWs and cement matrix in addition to the bridging of cracks. Overall, the thermal treatment increased the specific surface area of NWs enhancing their effects on cement properties, while SP improved the NWs dispersion, increasing their beneficial effects on the hardened properties.

## 1. Introduction

Nanomaterials are potential candidates to improve the mechanical properties and durability of cementitious composites. Their very high specific surface area can enhance the hydration of Portland cement by providing an extra surface for the nucleation and growth of hydration products, in addition to the potential binding activity of some nanomaterials [1,2]. In this context, the incorporation of several nanomaterials has been tested in cementitious matrices. Qing et al. [3] produced cementitious composites with nanosilica particles, observing a 25% increase in the 28-day compressive strength with the incorporation of 5 wt % of the nanomaterial. Garcia et al. [4] observed significant improvements in the durability of cementitious composite by adding 2 wt % of nanosilica, increasing the chloride binding ability and the refinement of the pore structure of the material. Andrade et al. [5] also reported durability improvements when added 3 wt % of nanosilica in the cementitious composite, observing a compressive strength increase of 44% and an average pore size reduction of 66% at 91 days for the nanosilica-containing composite in comparison with the plain mix. One of the most studied nanomaterials used to reinforce cementitious composites is carbon nanotubes (CNTs). In addition to the extra surface provided by the nanomaterial, CNTs can improve the mechanical performance of the composite by bridging across cracks and voids [6,7]. In this regard, Tyson et al. [8] produced cementitious composites with 0.1 and 0.2 wt % addition of CNTs, observing improvements in mechanical strength, ductility, and fracture toughness with the incorporation of the nanomaterial. For instance, the peak of displacement at failure (in the stress vs. strain curve of the mechanical strength test) increased by 150% while the 28-day flexural strength increased by 36% with CNTs addition. Li et al. [9] observed increases in the 28-day compressive and flexural strengths of 19% and 25%, respectively when added 2 wt % CNT in Portland cement paste. Abu Al-Rub et al. [10] produced cementitious composites with the 0.04–2.0 wt % addition of two types of CNTs, finding that the flexural strength increased by up to 269% while the ductility increased by up to 86% compared with plain cement paste at 28 days.

Another potential candidate for nanoreinforced cementitious matrix is SiC nanowhiskers (NWs), also called nanorods. This material usually presents exceptional mechanical properties, with a Young’s modulus of around 650 GPa and a mechanical strength of 50–55 GPa [11]. Compared with the most popular nanoreinforcement material (i.e., CNTs), SiC NWs presents some advantages. The hydrophilic nature of SiC NWs [12] makes them more easily dispersed in water and consequently in the cementitious matrix, in contrast to the hydrophobic CNTs. In addition, these NWs can hinder the growth and activity of bacteria [13], which is attractive for self-cleaning applications [14] such as self-cleaning concrete [15]. Finally, SiC NWs can be synthesized from simple organic raw materials, such as sorghum leaves [16], macroalga [17], and bleached wood pulp [18].

SiC NWs have already been incorporated into different types of matrices. Rahman and Al Rashed [19] incorporated 5–20 wt % SiC NWs in aluminum matrix composites, observing progressive improvements in their mechanical performance as the incorporation level increased, reaching 85% higher Vickers hardness and 173% higher tensile strength values for the highest incorporation level in comparison with the plain matrix. Du et al. [20] produced geopolymeric composites with 1–10 wt % addition of SiC NWs, reaching a 28-day compressive strength of 155 MPa, corresponding to a 100% enhancement compared with the plain matrix. Shui et al. [21] successfully produced porcelain ceramics with 1–3 wt % SiC NWs incorporation. A few works were conducted regarding the incorporation of SiC NWs in Portland cement matrix. Azevedo et al. [22] incorporated 0.25–1.00 wt % of raw SiC NWs in cementitious composites, observing increases of 107% and 75% in the flexural strength of the composite, respectively at 7 and 28 days of hydration when compared with plain cement paste. Azevedo et al. [23] investigated the effect of thermal treatment of SiC NWs on the fresh and hardened properties of contentious composites without a superplasticizer. The authors found 28-day compressive and flexural strength improvements respectively of 43% and 50% when 0.50 wt % SiC NWs thermally treated at 500 °C were incorporated in comparison with plain cement paste. A major issue in using nanomaterials in cementitious composites is the proper dispersion of these nanoparticles, which has a strong trend for agglomeration [24,25]. In this regard, chemical admixtures (such as superplasticizers) can be used to enhance the dispersion of both cement and nanomaterial particles and thus improve their spatial distribution within the composite. In this context, to the best of our knowledge, there are no reports investigating the use of chemical admixtures in cementitious composites containing thermally treated SiC NWs.

Thus, this work investigated the use of a polycarboxylate-based superplasticizer as a dispersing agent to improve the dispersion of SiC NWs in the Portland cement matrix. For this purpose, composites with different contents of raw and thermally treated SiC NWs were produced. The rheological properties, cement hydration, flexural and compressive strengths at 7 and 28 days, water absorption, thermal conductivity, and microstructure of cement pastes were evaluated.

## 2. Materials and Methods

### 2.1. Materials and Mixes

**Figure 1:**
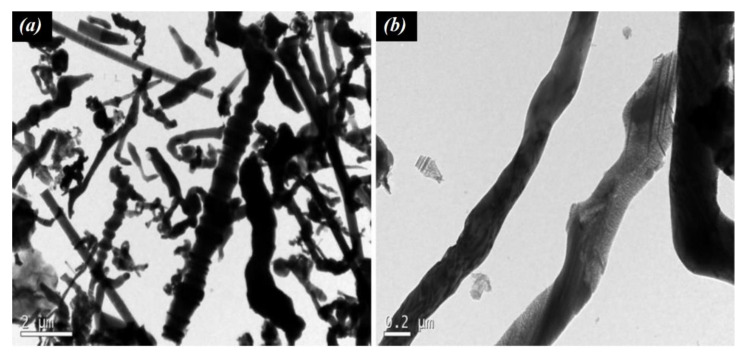
TEM images of the raw SiC NWs used. (**a**) 2 µm range, (**b**) 0.2 µm range.

The SiC NWs used in this work was supplied by Nanostructured & Amorphous Materials Inc. (Katy, TX, USA) and was composed of >99 wt % β-SiC, with a diameter of 0.1–2.5 µm, lengths of 2–50 µm, Blaine specific surface area (SSA) of 10.64 m^2^/g, density of 3.22 g/cm^3^, hardness of 9.5 Mohs, and free carbon content lower than 0.05 wt %. Figure 1 shows transmission electron microscopy (TEM) of the raw SiC NWs used, recorded using a JEM-1011 TEM (JEOL, Tokyo, Japan) microscope. The NWs were thermally treated at 500 °C in an electric oven for 1 h in order to remove any amorphous carbon of the NWs’ surface, resulting in a Blaine SSA of 11.51 m^2^/g and density of 6.05 g/cm^3^. In this study, the non-treated (i.e., as supplied) NWs are referred to as “NT-NW” while the thermally treated NWs are referred to as “500-NW”. The thermal treatment parameters were based on previous tests reported in [26].

**Figure 2:**
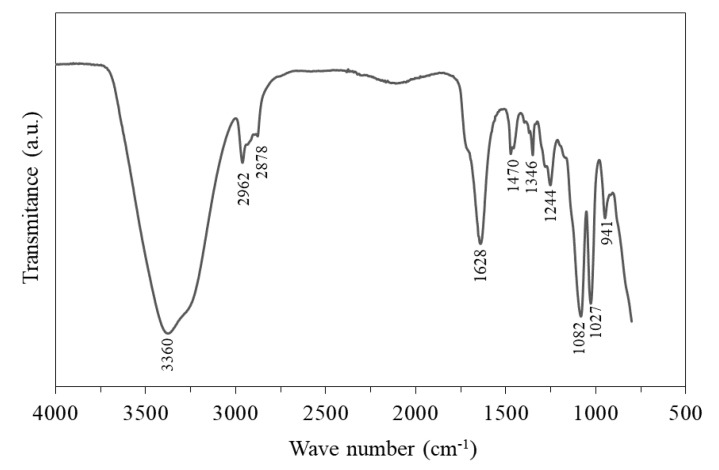
ATR-FTIR spectrum of the SP used.

Ordinary Portland cement (OPC) and polycarboxylate-based (PCE) superplasticizer (SP) were used for paste production. The detailed composition of the pastes investigated is presented in Table 1. The OPC used was equivalent to CEM I 42.5 R [27] and had a median diameter of 27 µm and Blaine SSA of 0.33 m^2^/g. The mineralogical composition of the cement used was determined by X-ray diffraction and Rietveld analysis and follows: 55.8 wt % C_3_S; 14.5 wt % C_2_S; 3.9 wt % C_3_A; 10.5 wt % C_4_AF; 2.8 wt % gypsum; 8.2 wt % calcite; about 4 wt % of minor phases (e.g., dolomite, periclase, and quartz). The SP had a density of 1.10 g/cm^3^, solid content of 51 wt %, and pH between 4.5 and 6.5. Figure 2 shows the attenuated total reflectance Fourier transform infrared spectroscopy (ATR-FTIR, Spectrum 3, PerkinElmer, Waltham, MA, USA) characterization of the SP, recorded using a Perkin Elmer (Frontier) equipment at the 4000–800 cm^−1^ range. The broad band at 3360 cm^−1^ and the narrow band at 1628 cm^−1^ are attributed to the vibrations of hydroxyl (–OH) of water. The bands at 2962, 2878, 1470, and 1346 cm^−1^ are attributed to C–H vibrations of alkane groups. The bands at 1244, 1082, and 941 cm^−1^ are respectively attributed to the C–O stretching of carboxylate groups, the –C–O–C– stretching of ether groups and the O–H bending of carboxylic acid. The band at 1698 cm^−1^ corresponds to the C=O stretching of carboxylic groups and the band at 1027 cm^−1^ corresponds to the C–N stretching vibration of amine groups.

### 2.2. Sample Preparation and Testing Methods

SiC NWs were previously dispersed in deionized water using ultrasonication for 10 min following [22]. Cement pastes with a constant water/cement ratio of 0.4 by weight were produced in a high-shear mixer, mixing the materials at 10,000 rpm for 3 min. Immediately after that, the isothermal calorimetry and rheometry tests started. For the calorimetry tests, a TAM Air (TA Instruments, Milford, MA, USA) calorimeter operating at 23 °C was used and the heat release was recorded up to 70 h. For the rheological tests, a HAAKE MARS III (Thermo Scientific) rheometer with concentric cylinders was used. The shear rate ($\dot{\gamma }$) was increased from 5 to 100 s^−1^ during 90 s and then decreased back to 5 s^−1^ in another 90 s, recording the shear stress (τ) applied. The rheological properties yield stress (τ_0_) and plastic viscosity (η_p_) were obtained by fitting the decreasing part of the flow curve with the Bingham model (Equation (1)).

τ = τ_0_ + η_p_· γ (1)

In addition, the sample spread was evaluated through the mini slump test [28]. The SP content used varied with the SiC NWs content, corresponding to the amount needed to obtain similar spreads of the reference paste in the mini slump test (see Section 3.1). All the fresh-state tests were conducted in two independent samples to account for test variability and mean values were adopted.

For hardened-state tests, the following properties and the respective number of specimens tested at each age were analyzed: compressive strength and water absorption (six cylindrical specimens of 20 mm in diameter × 40 mm in height), flexural strength (three prismatic specimens of 20 mm × 20 mm × 100 mm), and thermal conductivity (three specimens of 20 mm in diameter × 10 mm in height). The mechanical tests were conducted on an Instron Universal Testing Machine, while a C-Therm TCI equipment and a Wakefield thermal grease were used for the thermal conductivity tests. Finally, secondary electrons scanning electron microscopy (SE-SEM) was conducted in pieces of the specimens tested for compressive strength at 28 days, using a JSM-6701F (JEOL, Tokyo, Japan) microscope.

### 2.3. Statistical Analysis

A one-way ANOVA (α = 0.05) of the results of mini slump, rheological parameters and hardened state properties was performed in Origin Pro 8.6 software (Originlab, Northampton, MA, USA). It was assumed that data for each series had a normal distribution and the Tukey test was conducted.

## 3. Results and Discussion

### 3.1. Fresh-State Properties

**Figure 3:**
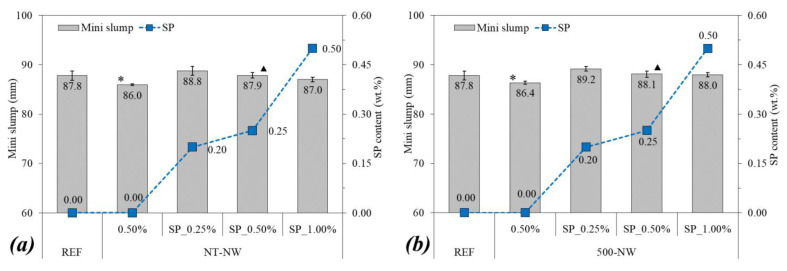
Mini slump and superplasticizer (SP) content of (**a**) NT-NW and (**b**) 500-NW-based systems. Stars indicate significant differences in comparison with REF and triangles indicate significant differences between the samples with and without SP.

Figure 3 shows the mini slump spread of the pastes and the SP content required to reach the target flowability, i.e., the mini slump spread of the REF mix ± 1.5 mm. The incorporation of 0.50% of either NT-NW or 500-NW alone (without SP) led to minor decreases in the mini slump of the pastes (1.8 mm for the former and 1.4 mm for the latter). In the systems with SP incorporation, the SP content required to reach the target sample spread increased as the NW content increased, from 0.20% of SP for 0.25% of either NW to 0.50% of SP for 1.00% of either NW.

**Figure 4:**
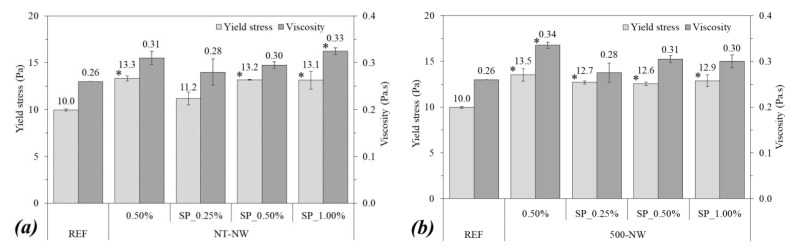
Yield stress (Pa) and plastic viscosity (Pa.s), determined by rheometry, of (**a**) NT-NW and (**b**) 500-NW-based systems. Error bars correspond to ±1 standard deviation. Stars indicate significant differences in comparison with REF and triangles indicate significant differences between the samples with and without SP.

Figure 4 shows the rheological properties of the pastes. The incorporation of 0.50% of NT-NW and 500-NW alone (i.e., without SP) increased the yield stress of paste by 33% and 35%, respectively, in comparison with the REF mix. Furthermore, the thermal treatment did not result in significant changes in the yield stress of paste, with a difference of 0.2 Pa between the yield stress of the NT-NW_0.50% and 500-NW_0.50% pastes. As for plastic viscosity, the incorporation of 0.50% NT-NW alone did not result in significant changes according to the statistical analysis, but the incorporation of 500-NT alone significantly increased the viscosity of paste (by 30.8%) compared with plain cement paste. Regarding SP incorporation, it led to a slight trend of reduction in both yield stress (from 13.3 Pa for NT-NW_0.50% to 13.2 Pa for NT-NW_SP_0.50% and from 13.5 Pa for 500-NW_0.50% to 12.6 Pa for 500-NW_SP_0.50%) and plastic viscosity (from 0.31 Pa.s for NT-NW_0.50% to 0.30 Pa.s for NT-NW_SP_0.50% and from 0.34 Pa.s for 500-NW_0.50% to 0.31 Pa.s for 500-NW_SP_0.50%). However, the Tukey test indicated that these differences were not statistically significant for a confidence level of 95%. Finally, although the mixes had comparable spreads in the mini slump test (86.0–89.2 mm, with a difference of up to 3.7%), the NW-containing pastes still had considerably higher yield stresses than plain cement paste (by up to 35% higher, with statistically significant differences) and this evidenced the limitation of the mini slump test to precisely describe the rheological behavior of cementitious mixes as previously discussed by [29].

Overall, the fresh-state tests indicated that the incorporation of NWs (either thermally treated or not) decreased the flowability of the pastes. This trend was associated with some factors: (i) the particle size of the NWs (much smaller than the cement) reduced the interparticle distance and increased the friction and the probability of collision between the particles, increasing the energy required to flow; (ii) the very high SSA of the NWs (10.64 m^2^/g for NT-NW and 11.51 m^2^/g for 500-NW compared with 0.33 m^2^/g for the cement) increased the surface interactions between the particles and consequently the trend for agglomeration; (iii) part of the mixing water was adsorbed on the surface of the hydrophilic NWs [12], reducing the amount of free water to lubricate the cement grains and increasing the relative particle concentration [30]; (iv) the rod-like shape of the NWs may hinder the relative movement between the grains. Similar results were reported by some authors when studying cementitious mixtures with NWs, CNTs, and carbon nanofibers [23,31,32,33].

### 3.2. Cement Hydration

**Figure 5:**
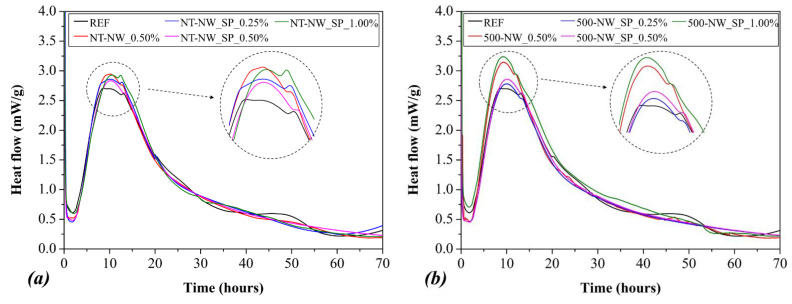
Heat flow of (**a**) NT-NW and (**b**) 500-NW pastes during the first 70 h of hydration. The insets highlight the main heat flow peak.

Figure 5 presents the heat flow curves (in mW/g of cement) of the cement pastes during the first 70 h of hydration. Comparing the pastes without SP, the addition of 0.50% NT-NW increased the main heat flow peak value by 9.0% while the addition of 0.50% 500-NW increased it by 16.2%. This enhancement in cement hydration probably happened because the nanomaterials provide an extra surface (i.e., nucleation sites) for C-S-H to grow, accelerating cement hydration, and was already reported in the literature for the incorporation of other nanomaterials [34]. Furthermore, the higher increase in the main heat flow peak for 500-NW compared with NT-NW can be related to the 8% higher SSA of the thermally treated NW (see Section 2.1), providing more surface for the nucleation and growth of the hydrated products.

Regarding the pastes with SP and NWs, the higher the NW content, the higher the increase in the main heat flow peak value, and this is associated with the increase in the surface area available for the nucleation and growth of the hydrated products discussed above. For instance, the mixes NT-NW_SP_1.00% and 500-NW_SP_1.00% presented the main heat flow peaks at 8.3% and 19.7% higher than the reference paste, respectively. However, when analyzing the effect of the SP incorporation (i.e., comparing NT-NW_0.50% vs. NT-NW_SP_0.50% and 500-NW_0.50% vs. 500-NW_SP_0.50%), one can note that the SP reduced the main heat flow peak value by 3.9% and 9.1%, respectively, for the NT-NW and 500-NW samples. Nonetheless, their main heat flow peak values were still higher than that of plain cement pastes. This agrees with previous reports [35,36,37,38], which observed delays in cement hydration when polycarboxylate-based SPs were added. According to some authors, this occurs because the molecules of SP adsorb on the cement particles’ surface. The adsorbed polymers both hinder the dissolution of anhydrous grains and reduce the surface area for the nucleation and growth of hydration products [35,36,37,39], consequently delaying the cement reactions. Some authors [37,39] related this retarding effect to the complexation of the Ca^2+^ ions in the solution by the carboxylate groups of PCE molecules, decreasing its concentration and delaying the supersaturation (which is known as the trigger for the end of the induction period and the start of portlandite precipitation [40]). However, according to Zhang et al. [35] and Winnefeld et al. [36], the Ca^2+^ complexation by PCE plays a negligible role in the retardation of cement hydration.

### 3.3. Hardened-State Properties

**Figure 6:**
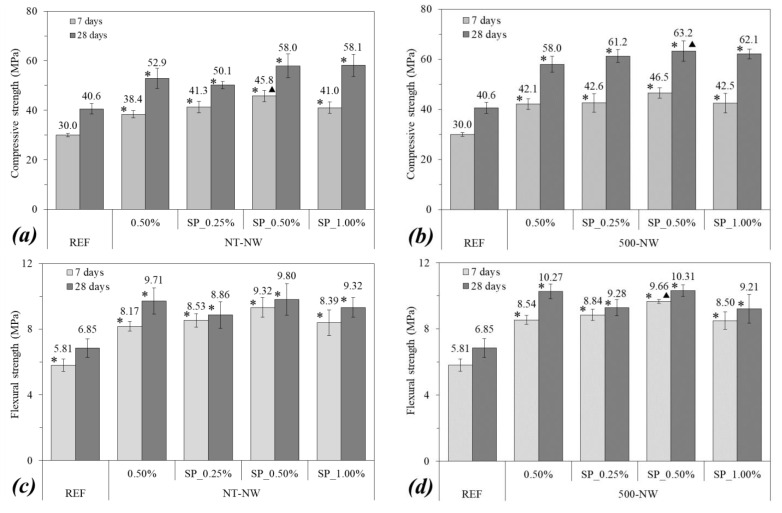
(**a**,**b**) Compressive strength; (**c**,**d**) flexural strength of the composites. Error bars correspond to ±1 standard deviation. Stars indicate significant differences in comparison with REF and triangles indicate significant differences between the samples with and without SP.

**Figure 7:**
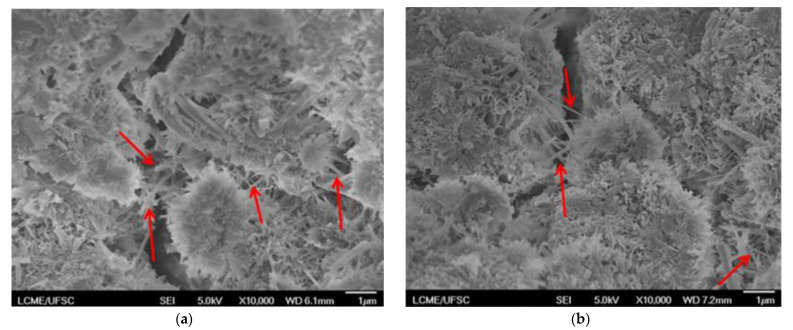
SE-SEM images (×10,000) of (**a**) NT-NW_SP_0.50% and (**b**) 500-NW_SP_0.50% composites at 28 days. The arrows indicate the NWs.

Figure 6 shows the compressive strength (a,b) and the flexural strength (c,d) of the cementitious composites at 7 and 28 days of hydration. The addition of 0.50% of NT-NW and 500-NW alone (i.e., without SP) resulted in 28-day compressive strength increases of 30% and 43%, respectively, and 28-day flexural strength increases of 42% and 50%, respectively, when compared with plain paste. The ANOVA/Tukey test indicated that these differences were statistically significant. These increase in mechanical strength with the addition of NW may be associated with some factors: (i) the filling of nanopores with the nanowhiskers can increase the compactness of the composite and therefore improve its mechanical strength [2]; (ii) the enhancement in cement hydration (indicated by the calorimetry results—Section 3.2) increases the amount of hydration products formed at a specific age, thus reducing the porosity of the composite; (iii) the so-called “bridge effect”, i.e., the NWs act as a nanoreinforcement, transferring the stress between the hydration products. This latter hypothesis is supported by the SE-SEM images of some NW-containing composites after the compressive strength test in Figure 7, which show that NWs can be found along the cracks, in line with previous reports for CNT-cement composites [6,8,9,10].

In general, the use of SP improved both the compressive and flexural strength of the composites. For instance, the NT-NW_SP_0.50% and 500-NW-SP_0.50% mixes presented compressive strengths from 9.0% to 19.3% higher than the respective mixes without SP. This probably happened due to the better dispersion of NWs with the SP, improving the effects (i) and (iii) discussed above. Comparing the mixes with SP and a different content of NWs, the 0.50% NW content showed the highest strengths for both NT-NW and 500-NW. This agrees with the results presented in [23], in which the 0.50% content led to the greatest mechanical performances for NW-cement composites without SP. This suggests that, despite improving the dispersion of NWs and consequently the mechanical performance composite, SP cannot properly disperse the NWs when this nanomaterial is used above the optimum incorporation level.

**Figure 8:**
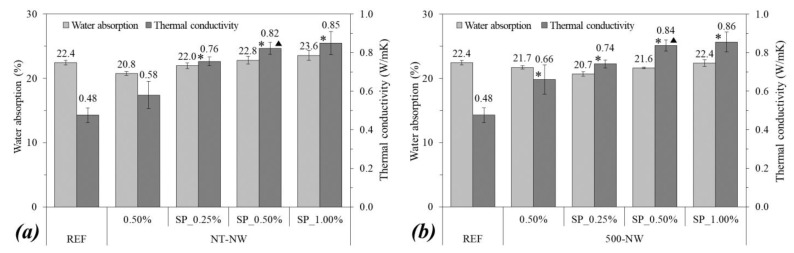
Water absorption and thermal conductivity of (**a**) NT-NWs cement composites and (**b**) 500-NWs cement composites, at 28 days of hydration. Error bars correspond to ±1 standard deviation. Stars indicate significant differences in comparison with REF and triangles indicate significant differences between the samples with and without SP.

Figure 8 shows the water absorption and thermal conductivity of (a) NT-NWs composites and (b) 500-NWs composites at 28 days of hydration. Regarding the water absorption, the addition of NWs did not lead to significant changes according to the statistical analysis, resulting in variations lower than 7.5%. One can note that those differences fall within the testing variability (error bars in Figure 8). This was expected since SiC NWs fill mainly the nanopores of the matrix (with a few nanometers in size) [31,41], which contribute little to capillary absorption, while the capillary voids, which reach up to 1 µm in size, are the main factor responsible for the water absorption and percolation in the cementitious matrix.

The addition of 0.50% 500-NW resulted in a significant increase (by 37.5%) in the thermal conductivity compared with the plain matrix. In turn, the addition of 0.50% NT-NW did not result in significant differences according to the statistical analysis. Regarding the mixes with SP, the higher the NWs content, the higher the thermal conductivity, reaching thermal conductivity values of 77.1% and 79.2% higher for NT-NW_SP_1.00% and 500-NW_SP_1.00%, respectively, compared with plain cement paste. These results agree with those reported by Du et al. [20], who observed increases in the thermal conductivity of geopolymer-SiC composites and this was expected since SiC whiskers are excellent heat conductors [20,42]. Similar results were also reported for the cementitious matrix reinforced with carbon nanotubes [43]. The higher increases in thermal conductivity with the addition of 500-NWs compared with NT-NWs are probably due to the higher surface area of the former.

The use of SP in the NWs-cement composites also increased the thermal conductivity values: the NT-NW_SP_0.50% mix presented a 41.4% higher value compared with the NT-NW_0.50% sample, and the 500-NW_SP_0.50% mix presented a 27.3% higher value compared with the 500-NW_0.50% sample. These increases were statistically significant according to the ANOVA/Tukey analysis. This can be explained by the higher dispersion of NWs in the presence of SP discussed above, enhancing their effect on the thermal conductivity of the cementitious composite.

## 4. Conclusions

In this work, cementitious composites were produced with a superplasticizer and both non-treated and thermally treated SiC NWs. SiC NWs incorporation slightly increased the yield stress of cement paste (by up to 10%) while it led to marginal effects in plastic viscosity (+0.02–0.05 Pa). NWs addition increased the main heat flow peak in calorimetry, indicating an enhancement in cement hydration in the first hours. NWs incorporation increased the compressive strength, tensile strength, and thermal conductivity of composites by up to 56%, 66%, and 80%, respectively, but it did not significantly affect the water absorptivity. The thermal treatment of SiC NWs increased their SSA, increasing the effects of NWs observed on the hydration and mechanical properties of the composites. The use of SP in NWs-containing samples led to a trend of reduction in both yield stress and plastic viscosity, but the differences were not statistically significant for a confidence level of 95%. Furthermore, SP incorporation delayed the early hydration of cement but increased the beneficial effects promoted by the NWs on the mechanical properties due to the better dispersion of the nanomaterial. SEM showed NWs placed along the cracks of the hardened matrix, confirming the bridging effect promoted by this nanoreinforcement, similarly to that reported in the literature for CNT-reinforced cementitious composites. These findings allowed us to conclude that SiC NWs are good candidates to the nanoreinforced cementitious matrix, increasing the mechanical performance of composite while not significantly impairing fluidity. In general, the coupled use of SP and thermal treatment improved the SiC NWs performance when added in the cementitious composite.

## Figures and Tables

**Table 1:** Detailed composition of the pastes investigated (by weight).

Mix	OPC	NT-NW	500-NW	Water	SP
REF	1.0	-	-	0.4	-
NT-NW_0.50%	1.0	0.50	-	0.4	-
NT-NW_SP_0.25%	1.0	0.25	-	0.4	0.20
NT-NW_SP_0.50%	1.0	0.50	-	0.4	0.25
NT-NW_SP_1.00%	1.0	1.00	-	0.4	0.50
500-NW_0.50%	1.0	-	0.50	0.4	-
500-NW_SP_0.25%	1.0	-	0.25	0.4	0.20
500-NW_SP_0.50%	1.0	-	0.50	0.4	0.25
500-NW_SP_1.00%	1.0	-	1.00	0.4	0.50

OPC: ordinary Portland cement; NT-NW: non-treated nanowhiskers; 500-NW: thermally treated nanowhiskers; SP: superplasticizer.

## Data Availability

Not applicable.
